# Miscellaneous

**Published:** 1891-10

**Authors:** 


### MISCELLANEOUS.

### SULPHURING OR BLEACHING DRIED FRUIT A MISTAKE IF NOT A CRIME.

(From the type of forthcoming volume of the American Public Health Association.)

BY JOEL W. SMITH, M. D., CHARLES CITY, IOWA.

The subject of this paper should command the careful attention of consumers of dried fruit, of conscientious fruit dealers, and of all health authorities. Fruit is now regarded more as a necessity than as a luxury, the want of it being a common cause of ill health.

As fresh fruit is not always obtainable, various methods for preserving it are in use, drying being one of the oldest and best for many fruits. Middle aged people recollect when sun or air drying was the only method for market. Then some good housewife discovered that more rapid drying by artificial heat, with or without the addition of sugar, was a cleaner method, safer against fermentation and decay, retained the flavor better, and the fruit was also lighter colored, than when sun or air dried. The present evaporators are only an enlargement of the idea of such more rapid drying, while canning consists of the exclusion of the microörganic germs of fermentation.

This is an age of progress, yet experience often shows that not all changes are improvements. It is about fifteen years since the sulphuring or bleaching of dried fruit began. At first only the uniform light color was sought, as in apples, pears, etc., but for some years past nearly all the large evaporating establishments have "sulphured" all kinds of fruits and some vegetables, and now much of the California sun-dried fruit for market is also treated in the same manner. The light color, especially of apples, early attracted unthinking consumers and commercial men, thus materially increasing the price of such fruit. That caused the practice to spread even to those who disapproved of it. The expense and trouble were very slight. Fruit so treated is said to dry more readily, consequently all now prefer to do it.

While the apparent change is only in color, there is a loss of the natural fruit flavor, even by the most careful sulphuring. Unfortunately some people do not notice the difference, but careful comparison shows it, as is admitted by the manufacturers of such fruit.

The practice began in California with apricots, as early as 1879. At the Twelfth State Fruit Growers' Convention, held in Fresno during four days in November, 1889, a paper on "Fruit Drying" was read by J. L. Mosher, of San Jose, and in his paper he remarked,—"If fruit be picked before ripe and over-sulphured to produce whiteness, it is devoid of its true rich taste and flavor, and *only requires polishing to make buttons.*" (The italics are his.) In discussing the paper, one gentleman said,—"I believe sulphuring the fruit is the greatest mistake in the world. I do it, but I believe it is wrong; the flavor of the fruit is gone after it is sulphured."

This change in quality was the first thing that called the attention of the writer's family to what was lacking in the "nice, uniformly colored" bleached fruits.

Later investigations have proved the presence of sulphate of zinc, "white vitriol," in all samples of fruit where zinc-surfaced trays were used to hold the sulphured fruit while drying. Interested parties have charged the German prohibition of American evaporated apples to rival trade opposition, but there is no German fruit to compete with them. The real cause was the finding zinc poison in considerable quantity. A good paternal government aims to protect its people. The advocates of sulphuring fruit say,—(1) It dries quicker, (2) looks better, (3) keeps better, and (4) sells better. Besides, it makes ripe, unripe and poor fruit all look alike, and if not so good for it, but few know it.

Sulphurous acid is formed by burning sulphur, and is readily absorbed by water. It abstracts oxygen from many vegetable substances, and thereby bleaches them. It also tends to prevent microscopic organizations that cause fermentation. The acid in liquid form is colorless, very cheap, and smells like burning sulphur; is antiseptic, a preservative fluid for some substances—sample fruits, etc. Sulphur is often burned to disinfect sick rooms of disease germs, and to kill rats, mice and vermin, but its use with food is objectionable. Ants and other insects, it is said, will not touch sulphured fruit, while they readily attack well ripened fruit that is not sulphured. The instinct of insects and animals is sometimes better than the practice of human beings. In general, substances that repel such creatures are hardly safe for human food. The effect of consumption has seemed to be a decided falling off in demand among the more intelligent class of people. Retail dealers know that many who once used dried fruit extensively say, "Somehow we have lost our relish for it," and have almost ceased to use it since the craze for sulphuring fruits began. Fruit men say, "The public demands sulphured fruit, will pay more for it, and we will supply it." The public will yet show them that it can get its eyes open. As the green and canned fruit interests are the only permanent gainers by the sulphuring process, they are interested to have it continued. It is not easy to obtain a superior quality of unbleached fruit. In 1889 several retail grocers who understood the question, corresponded with parties evaporating apples, The reply was that " If an order for not less than twenty barrels was received at one time, apples would be furnished unbleached, otherwise not." The slightly yellowish brown color of unbleached dried fruit is an evidence of ripeness, good quality, and proper drying. The more rapid the drying the lighter will be the color, and the fruit will keep well if at once properly excluded from the air. When sulphured, the good, the poor and the unripe all look alike. Not so with the unbleached. No poor nor unripe fruit can make good dried fruit. The gain of sulphuring is always with the dealer, and not with the consumer. In preferring looks to quality, the people are often at fault. Public enlightenment will correct most dietetic errors. Good health is now sought by many, and will be by more in the near future, through correct living, rather than by the swallowing of drugs. And in that more excellent way, " in the good time coming," there will be no demand for sulphured and other drugged fruit among intelligent people. There is danger from fruit in metal cans, as is well known, and fresh fruit is frequently unobtainable, while both are often more expensive than dried fruits. Good unsophisticated dried fruits are always harmless. If green fruits are at times unobtainable, canned fruits dangerous, and a popular craze has rendered dried fruits also dangerous, what can the suffering public do ? It is between the alternative of using no fruit, or that which is injured or poisonous. Is the sulphuring of fruit a mistake or a crime ?

To correct the error, enlighten the people and prohibit injurious practices. Legal suasion only will stop it at present. The common schools in many states are required to teach the effects of alcohol and narcotics. Why not also include the effects of different foods ?

---

### HOW TO COOK A HUSBAND.

More than a decade ago, in the Baltimore Cooking School, the following recipe for " Cooking a husband so as to make him tender and good," was contributed by a lady, presumably of experience. We commend it to our lady readers.

A good many husbands are utterly spoiled by mismanagement. Some women go about it as if their husbands were bladders, and blow them up. Others keep them constantly in hot water; others let them freeze by their carelessness and indifference. Some keep them in a stew by irritating ways and words. Others roast them. Some keep them in pickle all their lives. It cannot be supposed than any husband will be tender and good managed in this way, but they are really delicious when properly treated. In selecting your husband you should not be guided by the silvery appearance, as in buying mackerel, nor by the golden tint, as if you wanted salmon. Be sure and select him yourself, as tastes differ. Do not go to the market for him, as the best are always brought to your door. It is far better to have none unless you will patiently learn how to cook him. A preserving kettle of the finest porcelain is best, but if you have nothing but an earthenware pipkin it will do, with care. See that the linen in which you wrap him is nicely washed and mended, with the required number of buttons and strings nicely sewed on. Tie him in the kettle by a strong silk cord called comfort, as the one called duty is apt to be weak. They are apt to fly out of the kettle and be burned and crusty on the edges, since, like crabs and lobsters, you have to cook them while alive. Make a clear, steady fire out of love, neatness and cheerfulness. Set him as near this as seems to agree with him. If he sputters and fizzes do not be anxious; some husbands do this till they are quite done. Add a little sugar in the form of what confectioners call kisses, but no vinegar or pepper on any account. A little spice improves them, but it must be used with judgment. Do not stick any sharp instruments into him to see if he is becoming tender. Stir him gently; watch the while, lest he lie too flat and close to the kettle, and so becomes useless. You cannot fail to know when he is done. If thus treated you will find him very digestible, agreeing nicely with you and the children, and he will keep as long as you want, unless you become careless and you set him in too cold a place.

### FOOD EXHIBITION.

A novel Food Exhibition is to be held at Madison Square Garden, under the auspices of the Food Manufacturer's Association, during the month of October, 1892, embracing every thing pertaining to the food economy, but only such articles as the exhibitors are willing to put their names upon and vouch for their genuineness, will be allowed to have a place.

DOCT. JOSEPH SIMMS, the renowned physiognomist and author, who has travelled the world over in search of facts connected with the interesting science of which he is, perhaps, the best living exponent, has at length made his permanent home in California, as superior in many respects, including climate and productiveness, to any other known region. A recent letter from the Doctor, published in the "Nevada City Daily Transcript," gives a glowing picture of the State of his adoption.

St. Louis, June 21, 1888.

For a long while I have been in the habit of prescribing fluid extract of viburnum prunifolium, in those painful, functional disorders of the uterus and appendages occurring in cases that come under my care for renal and vesical diseases. My results have been satisfactory. Of late, I have given the remedy in the form of DIOVIBURNIA, as prepared by a well-known St. Louis Pharmacist, and the results are equally good, perhaps better, and the method of administration vastly superior. JOHN P. BRYSON.

SENECA, Kans., Aug. 24, 1891.

HERBA VITA REMEDY CO.,

Dear Sirs:—Enclosed you will find fifty (50) cents, for which send to my address some Herba Vita tea. We have used the sample sent us and think it much better than the Garfield tea. Yours truly,

ETTA NORTON, Seneca, Kans.

The third Annual Meeting of the Tri-State Medical Association of Georgia, Alabama and Tennessee, will be held in Chattanooga, October 27, 28 and 29, at Turner Hall.

